# Drivers for Rift Valley fever emergence in Mayotte: A Bayesian modelling approach

**DOI:** 10.1371/journal.pntd.0005767

**Published:** 2017-07-21

**Authors:** Raphaëlle Métras, Guillaume Fournié, Laure Dommergues, Anton Camacho, Lisa Cavalerie, Philippe Mérot, Matt J. Keeling, Catherine Cêtre-Sossah, Eric Cardinale, W. John Edmunds

**Affiliations:** 1 Centre for the Mathematical Modelling of Infectious Diseases, Department of Infectious Disease Epidemiology, London School of Hygiene & Tropical Medicine, London, United Kingdom; 2 Veterinary Epidemiology, Economics and Public Health group, Department of Pathobiology and Population Sciences, The Royal Veterinary College, Hatfield, United Kingdom; 3 GDS Mayotte-Coopérative Agricole des Eleveurs Mahorais, Coconi, Mayotte, France; 4 Epicentre, Paris, France; 5 Centre de coopération internationale en recherche agronomique pour le développement (CIRAD) UMR ASTRE, Cyroi platform, Sainte Clotilde, La Réunion, France; 6 Institut National de Recherche Agronomique (INRA) UMR 1309 ASTRE, Montpellier, France; 7 Bureau de la Santé Animale, Direction Générale de l’Alimentation, Paris, France; 8 Université de La Réunion, Saint Denis, France; 9 Direction de l’Alimentation, de l’Agriculture et de la Forêt de Mayotte, Mamoudzou, France; 10 WIDER, Warwick University, Coventry, United Kingdom; 11 Life Sciences, Warwick University, Coventry, United Kingdom; 12 Mathematics Institute, Warwick University, Coventry, United Kingdom; University of California Berkeley, UNITED STATES

## Abstract

Rift Valley fever (RVF) is a major zoonotic and arboviral hemorrhagic fever. The conditions leading to RVF epidemics are still unclear, and the relative role of climatic and anthropogenic factors may vary between ecosystems. Here, we estimate the most likely scenario that led to RVF emergence on the island of Mayotte, following the 2006–2007 African epidemic. We developed the first mathematical model for RVF that accounts for climate, animal imports and livestock susceptibility, which is fitted to a 12-years dataset. RVF emergence was found to be triggered by the import of infectious animals, whilst transmissibility was approximated as a linear or exponential function of vegetation density. Model forecasts indicated a very low probability of virus endemicity in 2017, and therefore of re-emergence in a closed system (i.e. without import of infected animals). However, the very high proportion of naive animals reached in 2016 implies that the island remains vulnerable to the import of infectious animals. We recommend reinforcing surveillance in livestock, should RVF be reported is neighbouring territories. Our model should be tested elsewhere, with ecosystem-specific data.

## Author summary

Rift Valley fever (RVF) is an arboviral hemorrhagic fever affecting primarily livestock in Africa and in the Arabian Peninsula. The conditions leading to RVF emergence are not fully understood, mainly because of data scarcity. Applied to the island of Mayotte (our ecosystem under study), for which 12 years RVF serological dataset are available, and by using a mechanistic model, we demonstrate that RVF epidemics related mainly to the introduction of infectious animals. Our work confirms that anthropogenic factors, such as livestock movements, need to be accounted for in order to understand the epidemiology of this disease. Our model should be tested elsewhere, with ecosystem-specific data.

## Introduction

Rift Valley fever (RVF) is a major vector-borne, zoonotic, and hemorrhagic fever (*Phlebovirus*, Family *Bunyaviridae*) that severely affects human health, animal health and livestock production mainly in Sub-Saharan Africa [1–3]. Its potential for spread and emergence in current disease-free areas (e.g. Europe, United States of America) is of growing global concern. The emergence (or re-emergence) of a disease has been defined by Woolhouse and Dye (2001) [4] as an “increase [*in its incidence*] following first introduction into a new host population, or [*an increase in its incidence*] in an existing host population” following specific ecological changes, e.g. in anthropological factors, agricultural practices or climate [4–6]. Theoretically, the conditions leading to RVF emergence may result from a sudden increase in vector density, the availability of susceptible animals, and the presence of the virus. The virus could be newly introduced or already present locally; maintained in the mosquito population, or circulating at a low level in livestock or wild animal populations, although little evidence exists on the latter [7–9]. Due to little existing data on RVF, those mechanisms have not been fully quantified. Previous work on RVF emergence conducted for the Horn of Africa using ecological statistical modelling showed that above-average rainfall and vegetation density (Normalized Vegetation Difference Index, NDVI) over 3 to 4 months could lead to RVF re-emergence [10], but it did not seem to be always the case, especially in Madagascar and Southern Africa, where other factors, such as the movements of animals or the level of livestock susceptibility may also play a role [11,12]. Finally, although a range of mathematical models have been developed to study RVF, they looked mainly at RVF spread, that is once the virus is introduced [13] or they assessed the impact of vaccination strategies [14], and only few were fitted to data [15–17].

**Fig 1:**
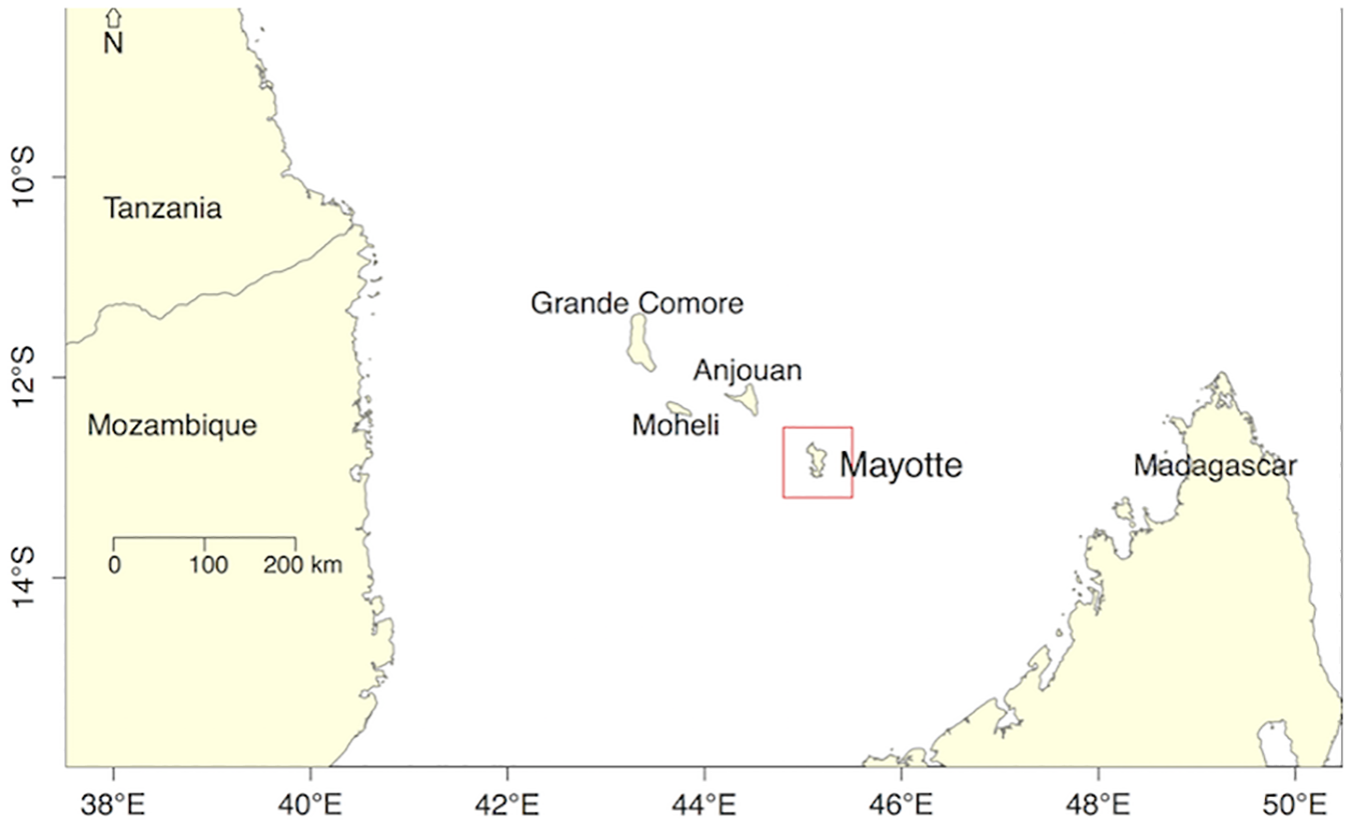
Location of the island of Mayotte. Mayotte is a small island located in the Mozambique Channel, between Madagascar and the African continent. Mayotte is a French department, while Grande Comore, Mohéli and Anjouan belong to the Union of the Comoros.

A large RVF epidemic started in Kenya in December 2006, affecting humans and animals, and subsequently spread to East and Southern Africa over the following months, including Somalia, Tanzania, Sudan, Mozambique, the Union of Comoros [10, 18–23]. In September 2007, RVF virus was detected in humans on the island of Mayotte (in the Mozambique Channel, Fig 1), about 500km away from the African continent. Two Mayotte 2008 RVF virus isolates were sequenced and the results showed that they were related to the Kenyan 2006–2007 clade [20,24]. Subsequent serological studies carried out in Mayotte in livestock (2004–2016) showed that RVF had been present at least since 2004, and re-emerged in 2008–10, despite no symptoms in animals being detected [25,26]. Because of its proximity to the Kenyan 2006–2007 clade, the Mayotte 2008 isolates may have been introduced onto the island by animal trade from the African mainland through the Union of Comoros [6,27]; and very likely resulted in the re-emergence observed in livestock in 2008–10 [25].

Quantifying the main factors driving pathogen emergence can be approached using mathematical models, but accounting for the diversity of the processes underlying emergence remains a challenge due to the lack of existing data [28]. Because of its insular nature, its epidemiological connections to the African mainland and its 12-year (2004–2016) RVF serological dataset [25], Mayotte offers an ideal setting to attempt to disentangle the impact of environmental and anthropogenic factors driving RVF dynamics in the livestock population. Here, we developed the first mathematical model for RVF emergence that accounts for livestock susceptibility, climate factors, and the import of infectious animals, while fitting serological data in a Bayesian framework. It allowed an estimate of (*i*) the most likely emergence scenario that could explain the past observed epidemic, and (*ii*) the likelihood of a future re-emergence, under different animal import scenarios.

## Methods

### Study area

Mayotte is a small island (374 km^2^), with an estimated livestock population size of about 30,000 heads [29] (17,000 cattle, 12,000 goats and 1,000 sheep). The production system is agro-pastoral; animals are raised for family consumption or ceremonies. No official export or import of animals exists. The movements of people on small boats (named "kwassa-kwassa") between Mayotte (French overseas *département*) and the nearest island of Anjouan (Union of Comoros), 70 km apart, became illegal since the installation of French visa requirements to enter Mayotte in 1995. Nevertheless, people attempt the journey with livestock on board [30]. Although the maritime border authorities attempt to control those entrants, they seize only a fraction of them (estimated 16,000 people entering per year in 2007 [31]), allowing the introduction of potentially infected animals.

The climate of Mayotte is marine tropical. The annual temperature varies between 25°C and 35°C, with a high annual rainfall (1500 mm) and a peak rainy season (December-March) [32]. Despite rainfall seasonality, the normalized difference vegetation index NDVI (a measure of vegetation density) is high and shows little annual variation (average Mayotte range 0.65–0.82, NDVI ranges 0–1) [33] (S1 Fig). A continually high NDVI potentially would allow the sustenance of mosquito breeding and therefore vector transmission even during the dry season [34,35]. Apart from RVF, other arboviral diseases reported are Dengue and Chikungunya [6].

### Model: Natural history of disease and demographics

The livestock population (cattle, sheep and goats) was modelled with an animal as a unit but without differentiating animals according to their species (one livestock population considered), or their spatial location. Animals could pass through four successive and mutually-exclusive infection states of RVF infection: Susceptible (S), Latent (E), Infectious (I) and Immune (R). Once infected, we assumed that animals remained in the (R) compartment, as natural infection is assumed to provide life-long immunity [14]. We accounted for deaths and births and assumed a constant population size (N) for the study period (October 2004-June 2016). The model was deterministic and discrete-time (weekly time step). While we assumed homogeneous mixing, the livestock population was purposively stratified in 10 yearly age groups *a* (*a* ∊[1–10]) to allow fitting the model to age-specific IgG prevalence data (see model fitting paragraph and S1 Text for details). The model is presented in S2 Fig, and in Eqs (1) to (7). Indexing the state variables and parameters by yearly age-group *a* (see S1 Text, methods section for the definition of age-groups), and time, *t*, we have the following equations:

For ≤ 12 months-old animals (i.e. age group *a* = 1):

$$S_{1,t+1}=(1-\lambda_{t})(1-\delta )\alpha S_{1,t}+b_{t}$$ (1a)

$$E_{1,t+1}=\lambda_{t}(1-\delta )\alpha S_{1,t}$$ (1b)

$$I_{1,t+1}=(1-\delta )\alpha E_{1,t}+I_{imp\_1,t}$$ (1c)

$$R_{1,t+1}=(1-\delta )\alpha R_{1,t}+(1-\delta )\alpha I_{1,t}$$ (1d)

For > 12 months-old to ≤ 108 months-old animals (i.e. age groups a ∊[2–9]):

$$S_{a,t+1}=(1-\lambda_{t})(1-\delta )\alpha S_{a,t}+(1-\lambda_{t})\delta \alpha S_{a-1,t}$$ (2a)

$$E_{a,t+1}=\lambda_{t}(1-\delta )\alpha S_{a,t}+\lambda_{t}\delta \alpha S_{a-1,t}$$ (2b)

$$I_{a,t+1}=(1-\delta )\alpha E_{a,t}+\delta \alpha E_{a-1,t}+I_{imp\_a,t}$$ (2c)

$$R_{a,t+1}=(1-\delta )\alpha R_{a,t}+\delta \alpha R_{a-1,t}+(1-\delta )\alpha I_{a,t}+\delta \alpha I_{a-1,t}$$ (2d)

For > 108 months-old animals (i.e. age group *a* = 10):

$$S_{10,t+1}=(1-\lambda_{t})\alpha_{10}S_{10,t}+(1-\lambda_{t})\delta \alpha S_{9,t}$$ (3a)

$$E_{10,t+1}=\lambda_{t}\alpha_{10}S_{10,t}+\lambda_{t}\delta \alpha S_{9,t}$$ (3b)

$$I_{10,t+1}=\alpha_{10}E_{10,t}+\delta \alpha E_{9,t}+I_{imp\_10,t}$$ (3c)

$$R_{10,t+1}=\alpha_{10}R_{10,t}+\delta \alpha R_{9,t}+\alpha_{10}I_{10,t}+\delta \alpha I_{9,t}$$ (3d)

With:

$$\lambda_{t}=1-\text{exp}(-\beta_{t}\sum_{a=1}^{10}I_{a,t})$$ (4a)

$$b_{t}=(1-\alpha )\sum_{a=1}^{10}(S_{a,t}+E_{a,t}+I_{a,t}+R_{a,t})-\sum_{a=1}^{10}I_{imp\_a,t}$$ (4b)

Where S_a_, E_a_, I_a_, R_a_, I_imp_a_, N_a_ are the age-specific number of Susceptible, Latent, Infectious, Immune, imported Infectious, and total number of animals in the *a*^*th*^ yearly age group, with their sum over all ages denoted by S_t_, E_t_, I_t_, R_t_, I_impt_ and N_t_, respectively. *λ*_*t*_ is the force of infection, and *β*_*t*_ is the per-capita effective contact rate. The rate at which latent (E) become infectious (I), and infectious (I) become immune (R) were fixed and equal to one, so that animals stay one time step (i.e. one week) in the (E) and (I) compartments. In the absence of vector data, the time spent in (E) is assumed to account for the extrinsic incubation period in the vector (3 days) and the latent (1–6 days) stage in the animal without explicitly modelling these processes, and the time spent in (I) accounted for the infectious stage in the host (3–6 days) [34,36,37]. This was chosen because we were interested in fitting the model to the Immune (R) compartment only, whilst allowing the serial interval (defined as the average time of infection between two consecutive cases, as per *Wallinga and Lipstich* [38] definition), at the animal level, being 2 weeks; which aligns with the 3 weeks estimated in South Africa at the farm level [39,40]. The rate at which animals are ageing at each time-step is noted δ; α is the survival rate for the age-groups 1–9, and α_10_ for the age-group 10; and finally *b*_*t*_ is the birth rate. No disease-related mortality was accounted for because such symptoms were not reported at that time in Mayotte, neither in the neighbouring Comoros and Mozambique RVF affected areas [23,41–43]. In addition, since the animal population was not fully susceptible at the beginning of our study period (October 2004), a proportion of immune animals (*imm_t*_*0*_) was specified at t_0_, such as:

$$R_{t=0}=N\times imm\_t_{0}$$ (5a)

$$S_{t=0}=N-(R_{t=0}+E_{t=0}+I_{t=0})$$ (5b)

### Climate-dependent transmission scenarios

We used NDVI as a proxy for climate conditions favouring mosquito habitat commonly used in RVF studies [10,11,44–46], as no data on vector dynamics was available. NDVI data for Mayotte did not show any three-months NDVI anomaly over the study period as measured for the Horn of Africa [10] therefore it was not used in this model (S1 Fig). Instead, we estimated the transmission parameter *β*_*t*_ that varied over time as a function of the observed NDVI value at time t (*NDVI*_*t*_) [33]. In the absence of a known quantified relationship between *β*_*t*_ and NDVI for RVF, or other vector-borne diseases, two models were tested. Model 1a assumed a linear relationship between *β*_*t*_ and *NDVI*_*t*_, and Model 1b an exponential relationship, such as:

$$\beta_{t}=\frac{R_{st}}{N}$$ (6a)

Model 1a:

$$R_{st}=a(NDVI_{t}-NDVI_{\text{min}})+b$$ (6b)

and Model 1b:

$$R_{st}=\text{exp}(aNDVI_{t}+b)$$ (6c)

Where *R*_*st*_ is the seasonal reproduction number, *a* and *b* are the coefficients of the linear and exponential functions. The linear function is defined such that *R*_*s*,*t*_ reaches its minimum value *R*_*smin*_ = *b* when *NDVI*_*t*_ is at its minimum (*NDVI*_*min*_).

### Virus introduction through animal imports

Following the RVF outbreak in the Horn of Africa in 2006–2007, it was assumed that infectious animals entered Mayotte in kwassa-kwassa, from a starting date *t*_*imp*_ and for a duration *P*. Those imported animals *I*_*imp*_ were added directly into the infectious compartments *I*_*a*_ (Eqs (1c), (2c) and (3c)), and at a constant flow at each time-step *t*, for the length of the period *P*, such as:

For

$$t_{imp}<\;t<\;t_{imp}\;+\;48P,\;I_{imp}=(n_{seized}p_{ikw})/(48p_{seized})$$ (7)

Where *n*_*seized*_ is the number of animals seized by the maritime patrol per year, *p*_*ikw*_ is the proportion of these that tested positive to RVF recent infection (S2 Table IgM positive), *p*_*seized*_ is the proportion of kwassa-kwassas seized, and finally *P* the duration of importation, expressed in year fraction. In addition, to facilitate the aggregation of monthly estimates, a month was modelled as 4 weeks, and therefore a year was 48 weeks. A time step (week) corresponded to 1.08 calendar week.

### Parameters

The parameters of the model were related to the natural history of disease and demographics, the climate-dependent transmission scenarios, or the viral introduction through animal import (Table 1). However, whilst some parameters were fixed input values, others were estimated by fitting the model predictions to the serological IgG prevalence data. Table 1 presents which parameters were fixed input values and which were estimated by fitting the model. Specifically, the fixed input parameters were the demographics parameters *N*_*a*_, α and α_10_ derived from demographic data [29,47,48] (S1 Text, S1 Table and S3 Fig), as well as the number of animals seized per year by the maritime patrol (*n*_*seized*_ = *100*), and the proportion of them being infectious (*p*_*kiw*_ = 15%) (S2 Table). The proportion of imported animals that had been recently infected by RVF virus (IgM positive, S2 Table) was used as a proxy for *p*_*kiw*_. The other six parameters were estimated by fitting the model to the serological data (Table 1) using a Bayesian framework. Each prior distribution of these parameters was a uniform function with the following lower and upper bounds: (*i*) the proportion of immune animals at *t*_*0*_, *imm_t*_*0*,_ could be estimated between 5 and 20%, (*ii*) *a* and (*iii*) *b*, the parameters defining the functional relationship between *βt* (and therefore *R*_*st*_) and NDVI were set to allow estimating *R*_*s*_ between 0.5 and 6, (*iv*) the proportion of kwassa-kwassas seized *p*_*seized*_ could take any value between 2.5% and 20%; and (*v*) the duration of import *P* varied from 1 month to 2 years. Finally, the date of the first import of infectious animals, (*vi*) *t*_*imp*_, could be any time between January 2007 (First report of RVF outside its initial confinement, i.e. in Kenya) and September 2007 (RVF detected in Mayotte), and was estimated from the data.

**Table 1:** Input fixed parameters and parameters to estimate with their input values range.

Parameter description	Notation	Values/distribution of the prior	Source
***Natural history of disease & demographics***			
Total population size	*N*	30,000	[29]
Prop. of immune animals at t_0_	*imm_t*_*0*_	Uniform [0.05,0.20]	to estimate by fitting the model to data
No. latent animals at t_0_	*E*_*0*_	5	-
No. infectious animals at t_0_	*I*_*0*_	5	-
Weekly ageing factor	*δ*	0.021 (1/48)/week	1month = 4 weeks in model
Survival rate for age-groups 1 to 9	α	0.9912/week	[47,48] and S1 Text
Survival rate for age-group 10	α_10_	0.9938/week	[47,48] and S1 Text
***Climate-dependent transmission scenarios***			
Model 1a: *Linear model*			
Slope	*a*	Uniform [1,6]	to estimate by fitting the model to data
R_s_ value at the minimum NDVI_t_ value	*b*	Uniform [0,1]	
Model 1b: *Exponential model*			
Multiplying factor	*a*	Uniform [1,20]	to estimate by fitting the model to data
Scaling factor	*b*	Uniform [–20,–1]	
***Viral introduction through animal import***			
No. of animals seized	*n*_*seized*_	100/year	S2 Table
Prop. imported infectious animals	*p*_*ikw*_	0.15/year	S2 Table
Starting date of animal import	*t_imp*	Uniform [Jan-Sept 07]	to estimate by fitting the model to data
Duration of import in years	*P*	Uniform [0.1,2]	
Prop. of kwassa-kwassas seized by the maritime border authorities	*p*_*seized*_	Uniform [0.025, 0.20]	

### Model fitting and parameter estimation

**Fig 2:**
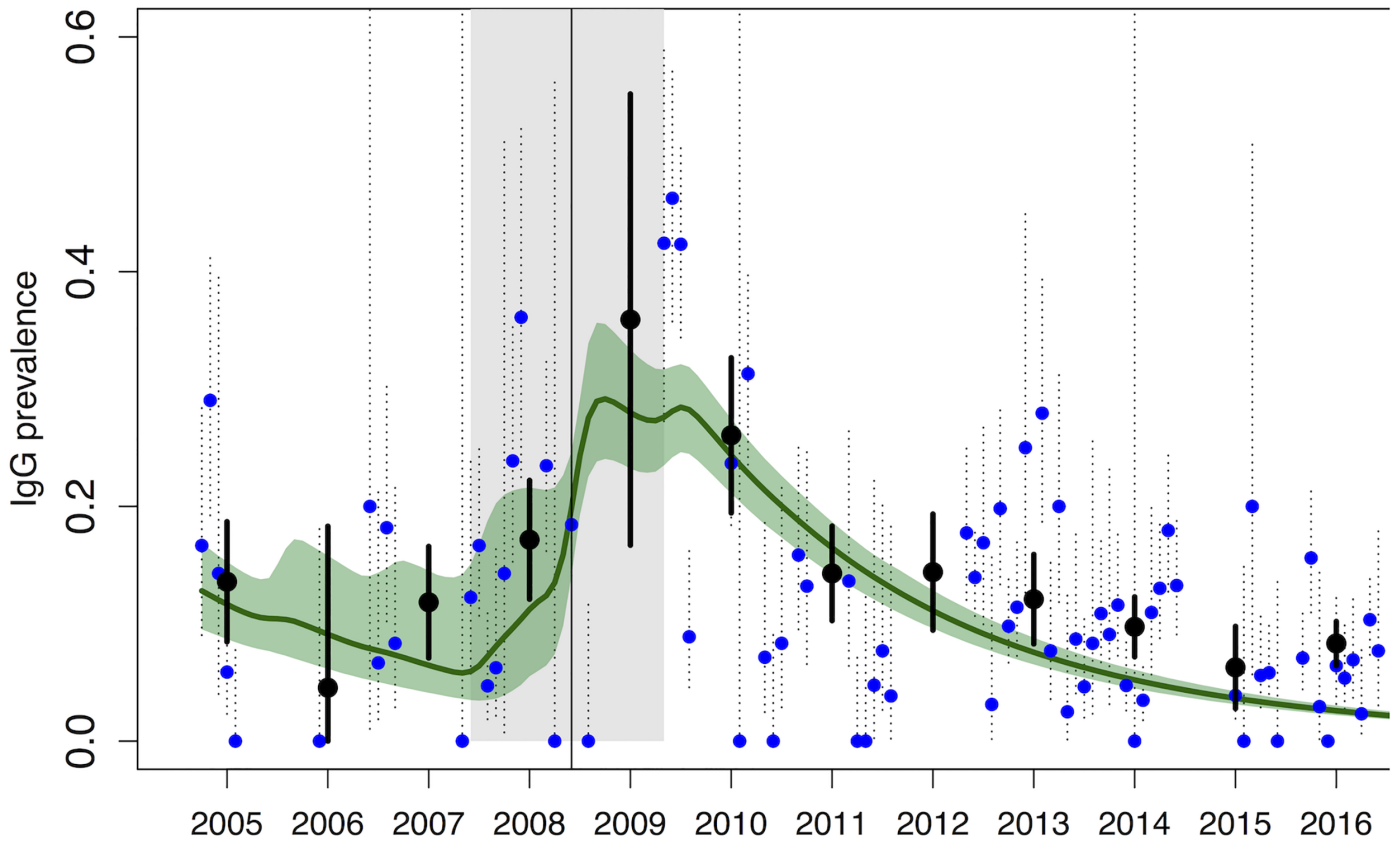
Model fit of the exponential model (Model 1b): Median (green line) and 95% CrI (green shaded area). Observed monthly (blue dots) and annual (black dots) IgG prevalence are shown, together with their 95% CI. For the period October 2004-June 2008 (before the vertical black line), the model was fitted to the monthly IgG prevalence (blue dots). For the period July 2008-June 2016, see Fig 3A–3H. The grey shaded area represents the estimated import period.

Parameter estimation was done by fitting the age-specific simulated proportion of immune (R) animals, for each epidemiological year *i*, to RVF IgG prevalence (Oct 2004-Jun 2016), as presented in Metras et al. 2016 [25]. Note that for Oct 2004-Jun 2008, age data was not available so we fitted to monthly prevalence (blue dots on Fig 2, S4 and S5 Figs). We sampled from the posterior distribution of all six parameters *θ = {imm_t*_*0*_,*a*,*b*,*t*_*imp*_,*P*,*p*_*seized*_*}* using a Monte Carlo Markov Chain Metropolis-Hastings algorithm [49], assuming uniform priors (Table 1). Parameters were estimated for both exponential and linear models (Models 1a and 1b), and the best model had the lowest deviance information criterion value (DIC) [50]. For details on parameter estimation, model fitting, and comparison see S1 Text.

### Model forecasting

To estimate the probability of re-emergence, we simulated 5000 stochastic trajectories of the exponential model, sampling randomly from the posterior distribution. The simulations were done for Oct 2004-Jun 2020, adding 48 months (Jul 2016-Jun 2020) to the original model, additional time for which the long-term NDVI monthly average values were used (however keeping a seasonal pattern). In “Forecast 1” infectious animals were only imported in 2007–09. To estimate the impact of future infectious imports and their seasonal timing on RVF re-emergence, we simulated the import of 1, 10, 20, 30 and 40 infectious animals in October 2016 (low NDVI values, Forecasts 2–6), and in April 2017 (high NDVI values, Forecasts 7–11).

### Sensitivity analysis

To test for the effects of animal imports on the probability of emergence, we fitted both linear (Model 2a) and exponential (Model 2b) models, without animal imports, therefore sampling from the posterior distribution of three parameters only *θ = {imm_t*_*0*_,*a*,*b}*. Finally, to assess the impact of NDVI seasonality on transmission, we fitted Model 3, a model with animal imports, but with a constant transmission parameter *β*, such as:

$$\beta_{}=\frac{R_{0}}{N}$$ (8)

In other words, we sampled from the posterior distribution of five parameters *θ = {imm_t*_*0*_,*β*,*t*_*imp*_,*P*,*p*_*ic*_}. All models were compared using the DIC [50].

### Ethics statement

The data were collected under the under a national disease surveillance system (Système d'Epidémiosurveillance Animale à Mayotte—SESAM) with the approval of the Direction of Agriculture, Food and Forestry (DAAF) of Mayotte. Before 2015, consent for blood sampling on a herd was obtained from its owner verbally after information in French (official language) or Shimaore (local language) was given. The animals were bled without suffering. No endangered or protected species were involved in the survey. From 2015, all procedures were approved by the London School of Hygiene Animal Welfare and Ethical Review Board.

## Results

**Fig 3:**
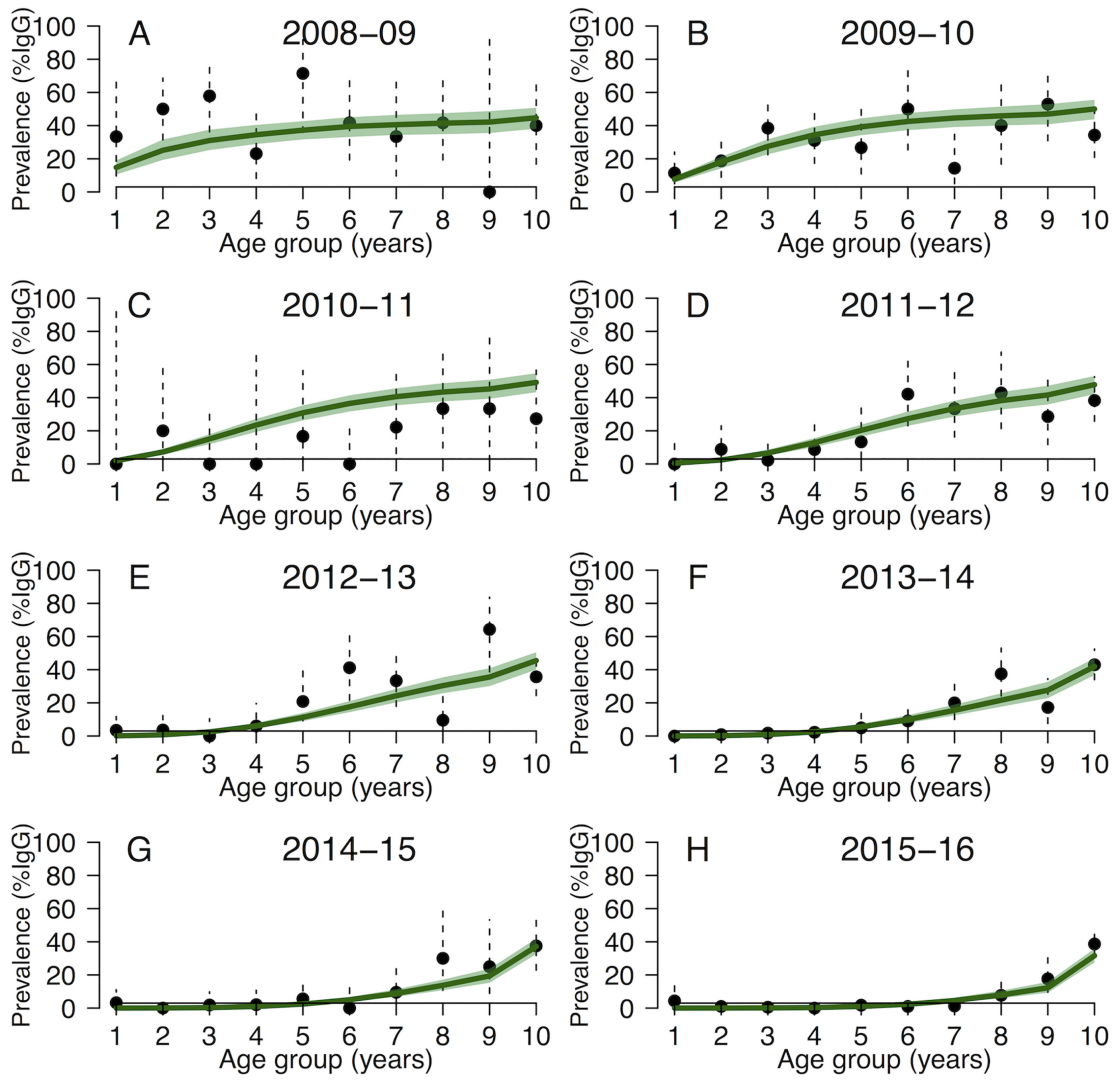
(A-H) Model fit of the exponential model (Model 1b) for (A-H) each epidemiological year between July 2008 and June 2016: Median (green line) and 95% CrI (green shaded area). The black dots are the observed annual age-stratified IgG prevalence (vertical dashed lines are the 95% CI).

Both linear and exponential models with seasonal variations of the NDVI including animal imports (Models 1a and 1b) fitted equally well the data (Table 2, *DIC*_*model1a*_ = 522.8 and *DIC*_*model1b*_ = 523.7) and showed a good agreement with the IgG serological data (Fig 2 and Fig 3A–3H). When transmission was not dependent on NDVI seasonality (Model 3), the IgG prevalence peak was also captured (S4 Fig, black solid line), but the model did not fit the data as well as the NDVI seasonality-dependent models, the DIC being higher (Table 2, *DIC*_*model3*_ = 543.6). Models without any animal import (Models 2a and 2b) had the worst fit (Table 2), and failed to capture the IgG prevalence peak (S4 Fig, blue and green solid lines), suggesting that the re-emergence of RVF virus in 2008–10 may have been due to the import of infectious animals.

**Table 2:** Deviance Information Criterions (DIC) for the five models tested, ordered from the best to the worst fit.

Model number	DIC	Model assumptions on	
		Animal imports	Transmission
Model 1a	522.8	Yes	seasonal NDVI, linear function
Model 1b	523.7	Yes	seasonal NDVI, exponential function
Model 3	543.6	Yes	no seasonal variation of NDVI (beta constant)
Model 2b	753.5	No	seasonal NDVI, exponential function
Model 2a	768.9	No	seasonal NDVI, linear function

Since both models 1a and 1b with animal imports exhibited a similar fit, we present in the main manuscript the fitting and forecasts using the exponential model (results obtained with the linear model are similar and provided in S5 Fig and S6A–S6H Fig). Fig 2 and Fig 3A–3H show the median and the 95% CrI of the 5000 stochastic trajectories of the proportion of IgG positive animals. The main discrepancies among model trajectories were observed for the first part of the study period (Oct 2004-Jun 2008, before the peak, Fig 2), when the model was fitted to serological estimates supported by a smaller sample size. In contrast, in July 2008-June 2016 the model was fitted to age-specific IgG prevalence (Fig 3A–3H), and simulations showed little variation.

**Fig 4:**
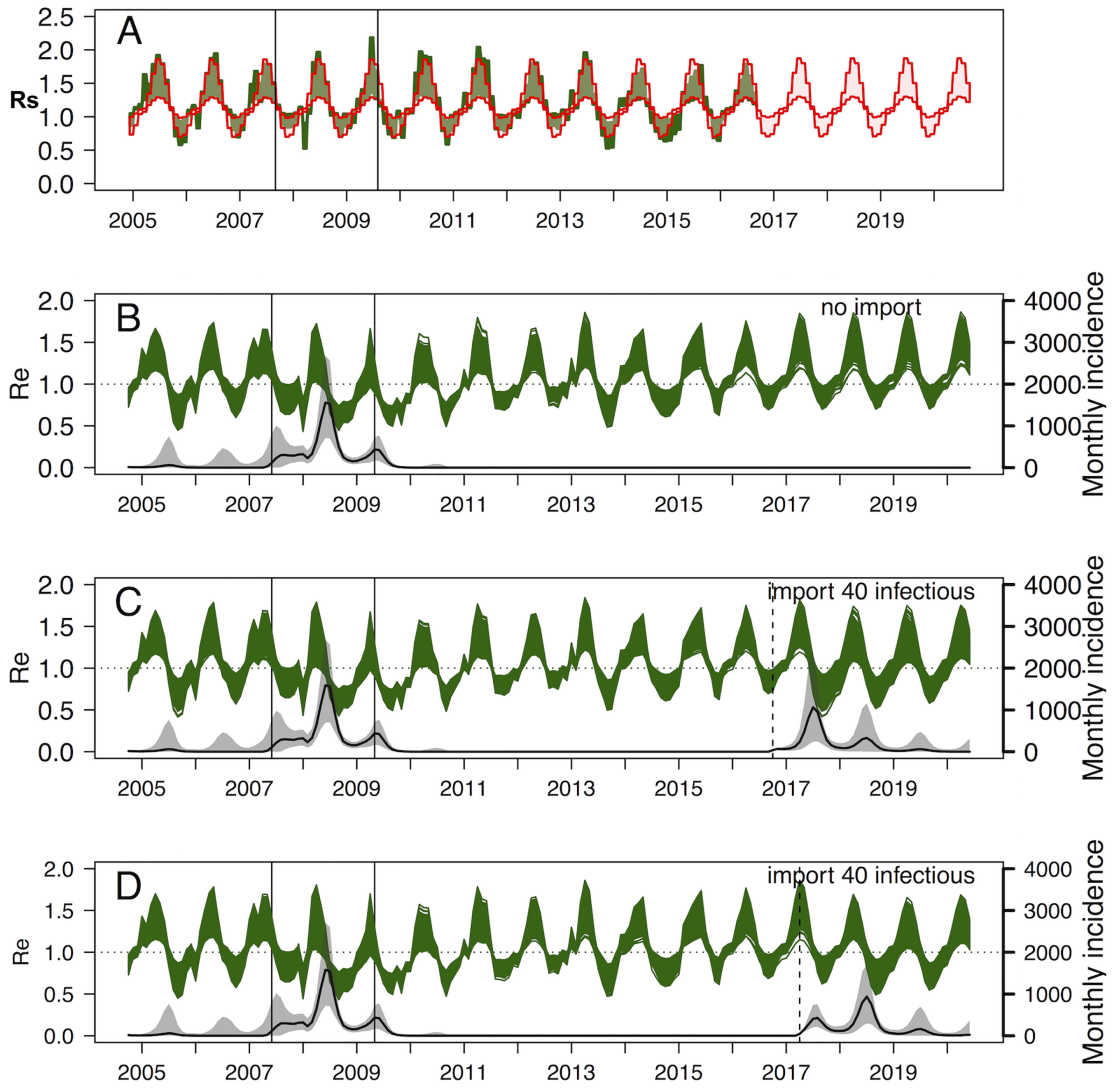
(A-D) Exponential model (Model 1b), (A) Seasonal variation of *R*_*st*_ (green area), reflecting the actual NDVI values and variation of *R*_*st*_ using long-term NDVI average values (red area). The period between the two vertical lines is the estimated import window. (B-D): Results of the stochastic forecasts: (B) Forecast 1: Effective reproduction number *R*_*e*_ over time (green lines, *R*_st_*proportion of susceptibles), and RVF incidence (solid black line) expressed as the number of newly infectious animals per month, together with their 95%CrI (grey shaded area), without import of infectious animals in 2016–17. (C) Forecast 6: with the import of 40 infectious animals in Oct 2016. (D) Forecast 11: with the import of 40 infectious animals in April 2017.

In the best models (Models 1a and 1b), the import of infectious animals was estimated to have started in *t*_*imp*_ = June 2007 (IQR [May 2007-Jul 2007], or month number 33), when 94.1% (IQR [85.6–96.5]) of the livestock population was estimated to be susceptible. The import scenario also estimated that 43 (IQR [39–46]) infectious animals were imported per month, during 23 months (IQR [22–24]), which corresponded to 2.9% (IQR [2.7–3.1]) of the animals illegally imported caught by the maritime border (Table 3). *R*_*st*_ values ranged between 0.36 and 1.90 for the linear model, and 0.52–2.19 for the exponential model (Fig 4A and S7 Fig), reflecting the seasonal variation of NDVI (NDVI_min_ = 0.59 and NDVI_max_ = 0.85). Finally, under those conditions, the proportion of immune animals at t_0_ (*imm_t*_*0*_), that is in October 2004, was 12.9% (IQR [11.7–14.1]).

**Table 3:** Median, interquartile range and 95% credibility interval of the six parameters estimated, and Deviance Information Criterions (DIC) for the two climate-dependent model scenarios (linear and exponential).

Scenario		Linear			Exponential		
DIC		522.8			523.7		
Parameters estimated	Notation	Median	IQR*	95%CrI^†^	Median	IQR*	95%CrI^†^
***Climate-dependent transmission scenarios***							
slope (linear) or multiplying factor (exponential)	a	3.41	3.02–3.80	2.28–4.56	3.15	2.75–3.57	2.05–4.41
*R*_*smin*_ (linear) or scaling factor (exponential)	b	0.67	0.62–0.72	0.52–0.81	-2.19	-2.50– -1.89	-3.11 – -1.38
*R*_*s*_ for maximum NDVI value (0.85)		1.55	1.30–1.90		1.91	1.39–2.19	
*R*_*s*_ for minimum NDVI value (0.59)		0.67	0.36–0.91		0.62	0.53–0.90	
***Initial conditions***							
Percentage of immune at t0	imm_t_0_	12.89	11.69–14.13	9.51–16.88	12.98	11.75–14.26	9.58–16.95
***Viral introduction through animal imports***							
Import starting date (month number)	t_imp	33.57	32.47–34.66	30.91–35.84	33.47	32.30–34.63	30.85–35.83
Import duration (year fraction)	P	1.88	1.79–1.94	1.59–1.99	1.88	1.78–1.94	1.58–1.99
Percentage of animals caught	p_seized_	2.86	2.66–3.14	2.51–3.82	2.99	2.72–3.37	2.52–4.42

* IQR: Interquartile range;

^†^ CrI: Credibility Interval

The seasonal variation of *R*_*st*_ over time reflected the seasonal NDVI values, and was compared to *R*_*st*_ values under the average-NDVI conditions (Fig 4A). The effective reproduction number *R*_*e*_ (range 0.42–1.84), and the monthly incidence (monthly number of infectious cases) are presented in Fig 4B. The incidence started to rise slightly in April-May 2007, that is before the import of infectious animals, and despite the imports starting in June 2007, the incidence remained stable and slightly decreased due to substantially below-average NDVI values. The highest incidence peak was reached the following year, in June 2008 (1558 cases, 95%CrI [707–2684]), and very likely resulted from the combination of infectious imports with above-average NDVI seasonal values; similarly to what is observed for the 2009 peak (Fig 4A). Following that import period in 2007–2009, the model predicted a very low probability of endemicity (Fig 4B, Forecast 1), with 99.74% of the trajectories indicating extinction in 2016 in a closed ecosystem. As of October 2016, 97.9% (95%CrI [97.6–98.2]) of the Mayotte livestock is estimated to be susceptible to RVF, such that the import of 40 infectious animals at that date (low NDVI values, Fig 4C, Forecast 6) or in April 2017 (high NDVI values, Fig 4D, Forecast 11) would result in an incidence peak similar to 2008 (Forecast 6: Jul 2017: 1063 cases 95%CrI [242–2385]; Forecast 11: Jul 2018: 936 cases 95%CrI [297–1696]). However, if the number of infectious animals introduced into Mayotte is less than 40, then the incidence peak remains substantially higher if importations take place in April, compared to importations taking place in October (April: Forecasts 2–5 in S8A–S8D Fig and October: Forecasts 6–10 in S9A–S9D Fig).

## Discussion

Our work is the first dynamic mathematical model on RVF that accounts for climate and animal imports, and which is fitted to long-term epidemiological data [13]. The importance of livestock import was characterized as a major driver for RVF emergence, similarly to what has been described for Madagascar [9]. Our model narrowed the virus entry window in Mayotte to May-July 2007, which represents a plausible 6 to 4-months delay following the first RVF report in Kenya and Tanzania (Dec 2006 and Feb 2007) [10,18,19]. We also estimated the import of about 40 infectious animals per month over 23 months, which is possible back in 2007–10. In the absence of animal movement data and epidemic curve in neighbouring territories, we assumed constant entrant flows of animals. While the actual entrant flows may have varied with time, due to climatic or anthropogenic factors (such as political or economic factors), the proportion of boats seized may have also varied for the same reasons. Therefore, choosing a constant import flow was the least biased and most parsimonious option, that could be improved should better data be available.

Rainfall and temperatures are known to have an impact on the dynamics of vector populations, and RVF virus can be transmitted by a large range of vectors species with different bio-ecologies [2, 14]. The dynamics of rainfall and temperature may therefore result in a complex RVF vector multi-population dynamics for which no data are available in our case; and attempting to account for this without data would only increase the model uncertainty. In addition, studies on RVF vectors (*Culex pipiens* and *Aedes taeniorhynchus*) showed that temperature above 26°C increased virus transmission rates [51,52], while in Mayotte the average temperature varies between 25°C and 35°C [32], potentially allowing transmission year-round. If data on vector population dynamics were available, and if the ecosystem studied could bear cooler temperatures, both temperature and rainfall should be accounted for. Here, we used NDVI as a proxy for vector habitat and therefore vector density in common with many previous RVF studies did [10,11,44–46,53]. Since Mayotte has not reported any NDVI anomalies as in the Horn of Africa [10], using monthly NDVI was the most relevant parameter to use. Furthermore, no previous dynamic models have used RVF transmission as a direct function of NDVI [13], although NDVI is used in most spatial modelling works [10,11,44–46,53]. Our model allowed quantifying of a functional relationship between NDVI and transmissibility for RVF, with the highest *R*_*s*_ value being 2.19, falling within the range of previously estimated *R*_*0*_ at 1.19 (95%CI [1.18–1.21]) [54] in a theoretical endemic setting; or 1.18 (range 0.5–2.1) [55], and 1.17 (range [0–3.68]) [17] in an epidemic context. Finally, our model offers a benchmark for exploring RVF transmissibility without vector data, and should be tested in ecosystems with different NDVI dynamics.

The credibility intervals of the estimated parameters were relatively narrow, and impacted only on the variability of the trajectories observed in 2004–08, when monthly prevalence estimates were informed by a small number of sampled animals, generating large confidence intervals. Little information was available on how these samples were collected [25], which could bias the model results. However, these samples retrospectively analyzed were randomly selected from a bank of sera collected under the annual veterinary services prophylaxis campaign, which attempted to be representative of the livestock population. Both models did not reach the peak prevalences in 2008–09, and since these points corresponded indeed to recent infections (IgM positive animals) [25], a biased serological sample in the data collected remains the most plausible explanation. From July 2008 onwards, trajectories showed only very little variability, and indicated a very low probability of a future re-emergence in the absence of new viral introductions; which is consistent with previous modelling conducted for Mayotte [34]. Finally, after 2008, the model is fitted to the age-stratified IgG prevalence and is in good agreement with the observed IgG prevalence, even in the latest years (2015–2016). The overall observed IgG prevalence in 2016 which appears higher than the simulated one is an artefact which can be explained by a high number of animals sampled in the oldest age-group.

We assumed that animals were at equal risk of acquiring infection and becoming seropositive across species and age-groups. Indeed, in Mayotte all animals regardless of age and species are raised outdoors, and we therefore assumed that they were at equal risk of being exposed to mosquito bites. In addition, while some studies found differences in serological prevalence between livestock species [56,57], a number of serological studies conducted in different study areas across Africa, such as Mozambique, Senegal, Tanzania, Kenya and Madagascar, did not show any difference in seroprevalence between livestock species during an epidemic or inter-epidemic period [23,41,58–64]. Finally, due to the island’s small size and the limited spatial variation of the ecosystem [65]; but also since herds are small (about 5–7 animals) [25,29], and herds from all communes had been affected by RVF, we did not need to account for spatial heterogeneity. This also allowed implementing model fitting in a data-scarce environment. Stratifying per location would have resulted in data points supported by fewer samples, increasing uncertainty and precluding model fitting. In addition, since our livestock population did not experience the classical symptoms of RVF (waves of abortions and high mortality in newborn), disease-induced mortality was not explicitly modelled. Sub-clinical forms were common for RVF in Mayotte, as well as in the neighbouring Union of Comoros and Mozambique [23,41–43]. Also, sheep, the most susceptible species to clinical symptoms, only represents 3–4% of the livestock population of the island. Finally, in the absence of vector data, nor evidence on human-to-animals RVF transmission, we assumed that the import of infectious animals was the most likely virus introduction pathway, although the introduction through infectious vectors and infectious humans cannot be ruled out.

Model forecasts indicated a very low probability of RVF virus endemicity and therefore of re-emergence in a closed system. With a very high proportion of naive animals as reached in 2016, the livestock population remains vulnerable to the introduction of infectious animals. Since 2011, few RVF infections in Mayotte have been reported (few young RVF IgG positive animals, or IgM positive animals in 2013–15 [25], and no IgM positive in 2016), whilst the surveillance system has been strengthened over years, giving weight to our model results. Ongoing surveillance including both active (annual serological surveys), passive surveillance activities (reports of animal mortality and abortions by farmers), but also the strict control measures for illegally introduced animals (immediate euthanasia) are still currently in place in Mayotte. Given that the animal population is naïve, our results suggest that such surveillance must be maintained, and reinforced should RVF be reported in neighbouring territories. This includes raising farmers’ awareness to report mortality and abortion events, and mitigating the risk of human exposure through communication and preventive messages (best practices for abortion and raw meat handling, since most animals are still slaughtered at the farm with no individual protection equipment). Finally, assuming the availability of RVF, NDVI and animal movement data, our model framework could be adapted to other ecosystems to refine the ecosystem-specific relative role of livestock susceptibility, animal movements and NDVI-related transmissibility on RVF dynamics.

## Supporting information

S1 Text Supporting information (Methods & Results). (PDF)

S1 Fig Monthly rainfall (solid blue line), normalized difference vegetation index (NDVI) values (solid green line), average NDVI values (dashed green line), % NDVI anomaly measured as percentage departure from mean ((NDVI_t_-NDVI_average_)/NDVI_average_)*100. The two vertical black dashed lines show September 2007, date when RVF Mayotte isolate was detected for the first time, and May 2007, estimated median most likely date of virus introduction on the island.
(TIFF)

S2 Fig SEIR age-stratified model diagram (10 yearly age-groups, with a∊ [2–9] on the diagram). The black arrows represent the state transitions within the same yearly age group, while the blue arrows also account for the ageing of animals. The dashed lines correspond to disease stage transitions. The red arrows are the import of infectious animals, and the transmission parameter *β*_*t*_ in green is driven by climate variables. Parameters and notations are presented in Table 1.
(TIFF)

S3 FigLivestock population age structure.The grey bars represent the number of animals per age group according to the data *n*_*a*_ [47,48]; and the numbers written are ${\bar{N}}_{a}$ the estimated number of animals in each age group used to parameterize the SEIR model (see S1 Text).(TIFF)

S4 Fig Results of the sensitivity analysis. Models’ fit for the linear model (Model 2a, blue line and 95%CrI) and exponential model (Model 2b, green line and 95%CrI) assuming no animal import but allowing seasonal variations of the NDVI; and model fit with animal import but assuming no seasonal variation of the NDVI (Model 3, black line and 95%CrI). Monthly (blue dots) and annual (black dots) RVF IgG prevalence are displayed.
(TIFF)

S5 Fig Model fit (linear model 1a). Mean (solid blue line) and 95% CrI (blue shaded area). The model was fitted to the monthly seroprevalence (blue dots) for the period October 2004-June 2008 (left of the vertical black line). For the period July 2008-June 2016, the age-specific fit for each epidemiological year is shown in S6A–S6H Fig.
(TIFF)

S6 Fig (A-H) Model fit (linear model 1a). Model fit for July 2008-June 2016. The model was fitted to the observed annual age-stratified seroprevalence (black dots) for each epidemiological year.
(TIFF)

S7 Fig Results of Models 1a and 1b: Linear (blue shaded area) and exponential (green shaded area) relationships between NDVI (x-axis) and R_s_ (y-axis). *R*_*s*_ values range from 0.36 to 1.90 for the linear model, and 0.52 to 2.19 for the exponential model; for NDVI values varying between 0.59 and 0.85.
(TIFF)

S8 Fig (A-D) Additional forecasts for Model 1b (exponential model), assuming infectious imports in April 2017. Effective reproduction number *R*_*e*_ over time (green solid lines, *R*_s_*proportion of susceptibles), and median monthly incidence (solid black line) expressed as the number of newly infectious animals per month, with their 95%CrI (grey shaded area). (A) Forecast 2: import of 1 infectious animals, (B) Forecast 3: import of 10 infectious animals, (C) Forecast 4: import of 20 infectious animals, and (D) Forecast 5: import of 30 infectious animals.
(TIFF)

S9 Fig (A-D) Additional forecasts for Model 1b (exponential model), assuming infectious imports in October 2016. Effective reproduction number *R*_*e*_ over time (green solid lines, *R*_s_*proportion of susceptibles), and median monthly incidence (solid black line) expressed as the number of newly infectious animals per month, with their 95%CrI (grey shaded area). (A) Forecast 7: import of 1 infectious animals, (B) Forecast 8: import of 10 infectious animals, (C) Forecast 9: import of 20 infectious animals, and (D) Forecast 10: import of 30 infectious animals.
(TIFF)

S10 Fig (A-F) Example of autocorrelation plots for the 6 parameters of the MCMC chain (for Model 1b). (A) multiplying factor *a*, (B) scaling factor *b*, (C) proportion of immune at t0 *imm_t0*, (D) date of import *t_imp*, (E) proportion of boats seized *p_seized*, (F) duration of imports *P*.
(TIFF)

S1 Table Demographic parameters estimated in equations S1-S8 and used in the SEIR model. (PDF)

S2 Table Animal illegally entering and seized by the maritime borders in 2008 in Mayotte, and results of ELISA IgM testing (Data Veterinary Services, 2008). (PDF)
