# Supplementary material for: Drivers for Rift Valley fever emergence in Mayotte: A Bayesian modelling approach
Source: PLoS Negl Trop Dis. 2017 Jul 21;11(7):e0005767. doi: 10.1371/journal.pntd.0005767 (PMC5540619; doi:10.1371/journal.pntd.0005767)
Supplement: S1 Text — (PDF) [file pntd.0005767.s001.pdf]

S1 Text. SI Methods & Results

Drivers for Rift Valley fever emergence in Mayotte: a Bayesian modelling approach

Raphaëlle Métras[1*], Guillaume Fournié[2], Laure Dommergues[3], Anton Camacho[1,4], Lisa Cavalerie[5,6,7,8], Philippe Mérot[9], Matt J Keeling[10,11,12], Catherine Cêtre-Sossah[5,6] Eric Cardinale[5,6,&] & W John Edmunds[1,&]

[&]These authors contributed equally to this work

SI Methods

Natural history of disease and demographics. The age-stratified SEIR model diagram is presented in S2 Fig.

Parameters: survival rates using demographic data. In the absence of disease, the demographic model is:

For the zero-to-one year old animals (i.e. age group a=1):

$$N_{1,t+1} = \alpha(1-\delta)N_{1,t} + (1-\alpha)\sum_{a=1}^{9} N_{a,t} + (1-\alpha_{10})N_{10,t} \quad \text{(S1)}$$

For the one to nine years old animals (i.e. age groups a $\in$[2-9]):

$$N_{a,t+1} = \alpha(1-\delta)N_{a,t} + \alpha\delta N_{a-1,t} \quad \text{(S2)}$$

For animals of 9 years-old and older (i.e. age group a=10):

$$N_{10,t+1} = \alpha_{10}N_{10,t} + \alpha\delta N_{9,t} \quad \text{(S3)}$$

Age-groups presented in the above equations and S3 Fig were defined as follow:

Age-group 1 (a=1): Animals ≤ 12 months

Age-group 2 (a=2): > 12 months-old to ≤ 24 months-old

Age-group 3 (a=3): >24 months-old to ≤ 36 months-old

Age-group 4 (a=4): >36 months-old to ≤ 48 months-old

Age-group 5 (a=5): > 48months-old to ≤ 60 months-old

Age-group 6 (a=6): >60 months-old to ≤ 72 months-old

Age-group 7 (a=7): > 72 months-old to ≤ 84 months-old

Age-group 8 (a=8): > 84 months-old to ≤ 96 months-old

Age-group 9 (a=9): > 96 months-old to ≤ 108 months-old

Age-group 10 (a=10): > 108 months-old

Where δ is the weekly ageing factor (S1 Table), and α and α_{10} are the survival rates for age groups 1 to 9 and age-group 10, respectively. N_a is the number of animals in each age group ($a \in$[1-10]) (S3 Fig - with the number of small ruminants and cattle in each age group being summed up), and the population size N is equal to 30,000 and assumed constant:

$$N = \sum_{a=1}^{10} N_a \qquad \text{(S4)}$$

At the stable equilibrium, the number $\overline{N}_a$ of animals in each age group a can be expressed as a function of the population size N, δ, α and α_{10}:

$$\overline{N}_1 = \frac{N}{\theta} \text{ with } \theta = 1 + \sum_{x=1}^{8}\left[\frac{\delta\alpha}{1-(1-\delta)\alpha}\right]^x + \left[\frac{\delta\alpha}{1-\alpha_{10}}\right]\left[\frac{\delta\alpha}{1-(1-\delta)\alpha}\right]^8 \qquad \text{(S5)}$$

$$\overline{N}_{10} = \left[\frac{\delta\alpha}{1-\alpha_{10}}\right]\left[\frac{\delta\alpha}{1-(1-\delta)\alpha}\right]^{8}\frac{N}{\theta} \qquad \text{(S6)}$$

And for $a \in [2\text{-}9]$:

$$\overline{N}_{a} = \left[\frac{\delta\alpha}{1-(1-\delta)\alpha}\right]^{a-1}\frac{N}{\theta} \qquad \text{(S7)}$$

N and δ are fixed, N=30,000 and δ=0.021, and the parameters α and α_{10} are estimated by maximising a Poisson likelihood function: the observed number of animals n_a in each age-group a follows a Poisson distribution of mean $\overline{N}_a$, $n_a \sim Poisson(\overline{N}_a)$, and the likelihood function is expressed as:

$$L = \prod_{a} \frac{\overline{N}_a^{\,n_a} e^{-\overline{N}_a}}{n_a!} \qquad \text{(S8)}$$

The estimated values of α and α_{10} are shown in S1 Table. The reported n_a and simulated number of animals at equilibrium $\overline{N}_a$ of animals in each age category are both shown in S3 Fig.

Parameters. S2 Table shows the results if the IgM testing on imported animals caught in 2008.

Model fitting and parameter estimation. Parameter estimation was done by fitting the age-specific simulated proportion of immune animals $p_{a,i}$, for each epidemiological year i, to RVF serological data (Oct 2004-Jun 2016), as presented in Metras et al. 2016 [25], such as (black dots on Fig 3A-H and S6A-H Fig):

$p_{a,i} = R_{a,i}/N_a$, a in [1, 10] (S9)

Note however that for Oct 2004-Jun 2008, serological data were only available for the whole population so we aggregated model outputs by month (blue dots on Fig 2, S4 Fig, and S5 Fig):

$$p_i = \sum_{a=1}^{10} p_{a,i} \qquad \text{(S10)}$$

We sampled from the posterior distribution of all six parameters $\theta = \{imm\_t_0, a, b, t_{imp}, P, p_{seized}\}$ using a Monte Carlo Markov Chain Metropolis-Hastings (MCMC-MH) algorithm [49]. For all parameters, we assumed uniform priors (Table 1). The number of IgG positive animals $x_{a,i}$ among the $n_{a,i}$ tested animals in the age-group a during period i followed a binomial distribution;

$$x_{a,i} \sim Bin(n_{a,i}, p_{a,i}) \qquad \text{(S11)}$$

The log-likelihood of the data was therefore given by:

$$l(data|\theta) = \sum_i \sum_a l_{a,i}(x_{a,i}, n_{a,i}|\theta) \qquad \text{(S12)}$$

$$l_{a,i}(x_{a,i}, n_{a,i}|\theta) = x_{a,i} \log p_{a,i} + (n_{a,i} - x_{a,i}) \log(1 - p_{a,i}) \qquad \text{(S13)}$$

We ran two independent MCMC-MH chains of 100,000 iterations, and visually checked that both chains converged to the same stationary distribution. We discarded the first 5,000 iterations of the burn-in periods and thinned at a ratio of 1:20 to eliminate auto-correlation (S10A-F Fig), finally we combined both chains. The median, 95% Credible Interval (CrI) and interquartile range (IQR) of the posterior distributions were computed (Table 3). The best model among the two had the lowest deviance information criterion value (DIC) [50]:

$$DIC = D(\theta_m) + 2p_D \qquad \text{(S14)}$$

$$D(\theta) = -2l(data|\theta) + k \qquad \text{(S15)}$$

$$p_D = \frac{1}{2}\mathrm{var}(D(\theta)) \qquad \text{(S16)}$$

Where $D(\theta)$ is the deviance of the model for a parameter set θ, $l(data|\theta)$ is the likelihood of the data given θ, and k is a constant that cancel out when comparing models; θ_m is the posterior mean of θ and p_D is the effective number of model parameters, computed as half the variance of the deviance under the posterior.

SI Results

S4 Fig shows the fit of both linear and exponential models with no import at all over the study period (models 2a and 2b), and of model 3 with NDVI not tied to transmission. S7 Fig presents the resulting relationships between NDVI and R_s for both the exponential and linear models. Both fits yielded very similar results (S5 Fig and S6A-H Fig compared with Fig 2 and Fig 3A-H). Finally, the Forecasts 2-5 and Forecasts 7-10 of the different scenarios of infectious animal imports (1, 10, 20, 30 infectious animals introduced in October 2016 and April 2017) are presented in S8A-D Fig and S9A-D Fig. S10A-F Fig presents the autocorrelation plots for the 6 parameters of a MCMC chain for Model 1b.
