# Supplementary figures and images for: Drivers for Rift Valley fever emergence in Mayotte: A Bayesian modelling approach
Source: PLoS Negl Trop Dis. 2017 Jul 21;11(7):e0005767. doi: 10.1371/journal.pntd.0005767 (PMC5540619; doi:10.1371/journal.pntd.0005767)

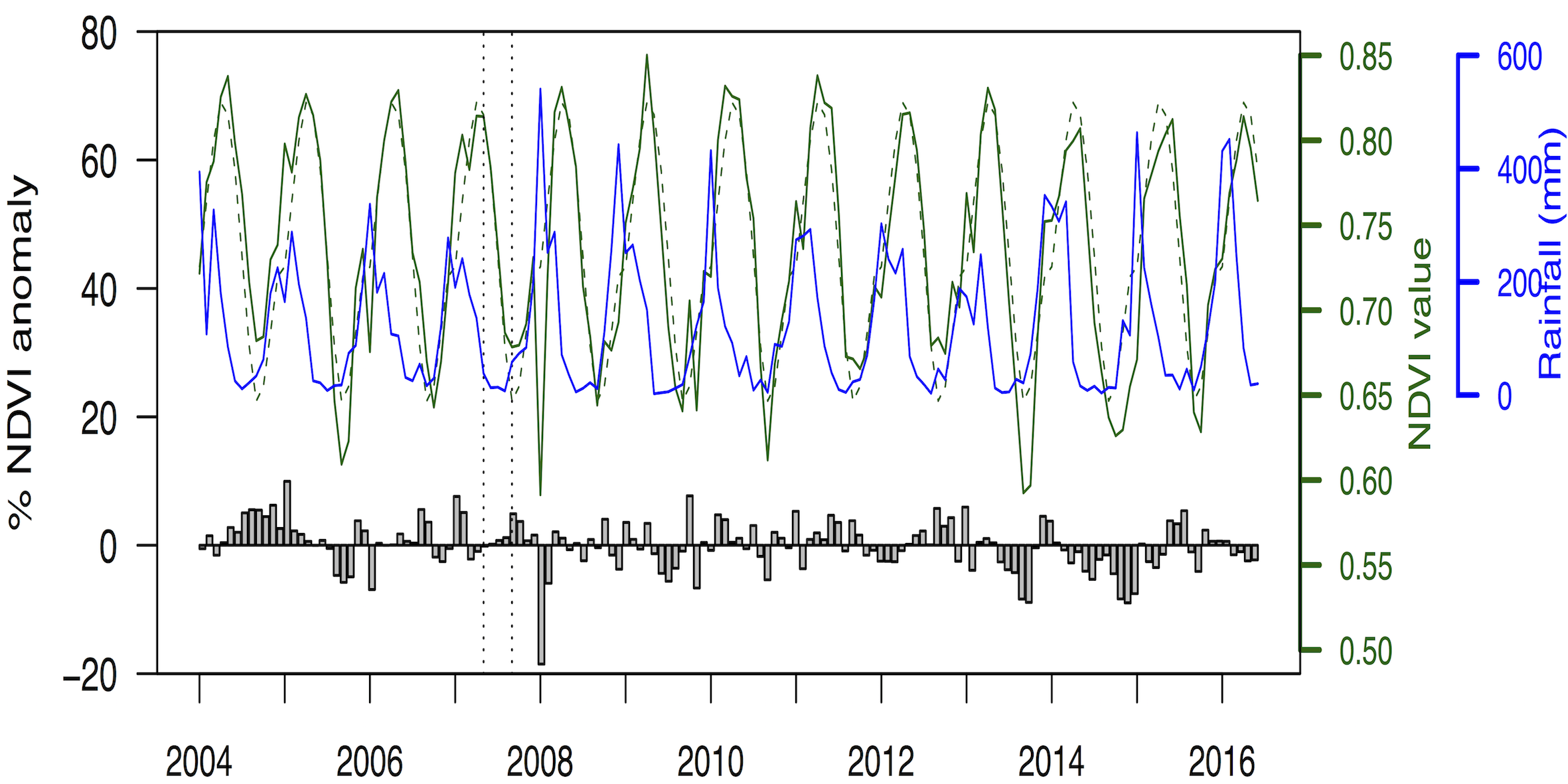

Supplement: S1 Fig — The two vertical black dashed lines show September 2007, date when RVF Mayotte isolate was detected for the first time, and May 2007, estimated median most likely date of virus introduction on the island. (TIFF) [file pntd.0005767.s002.tiff]

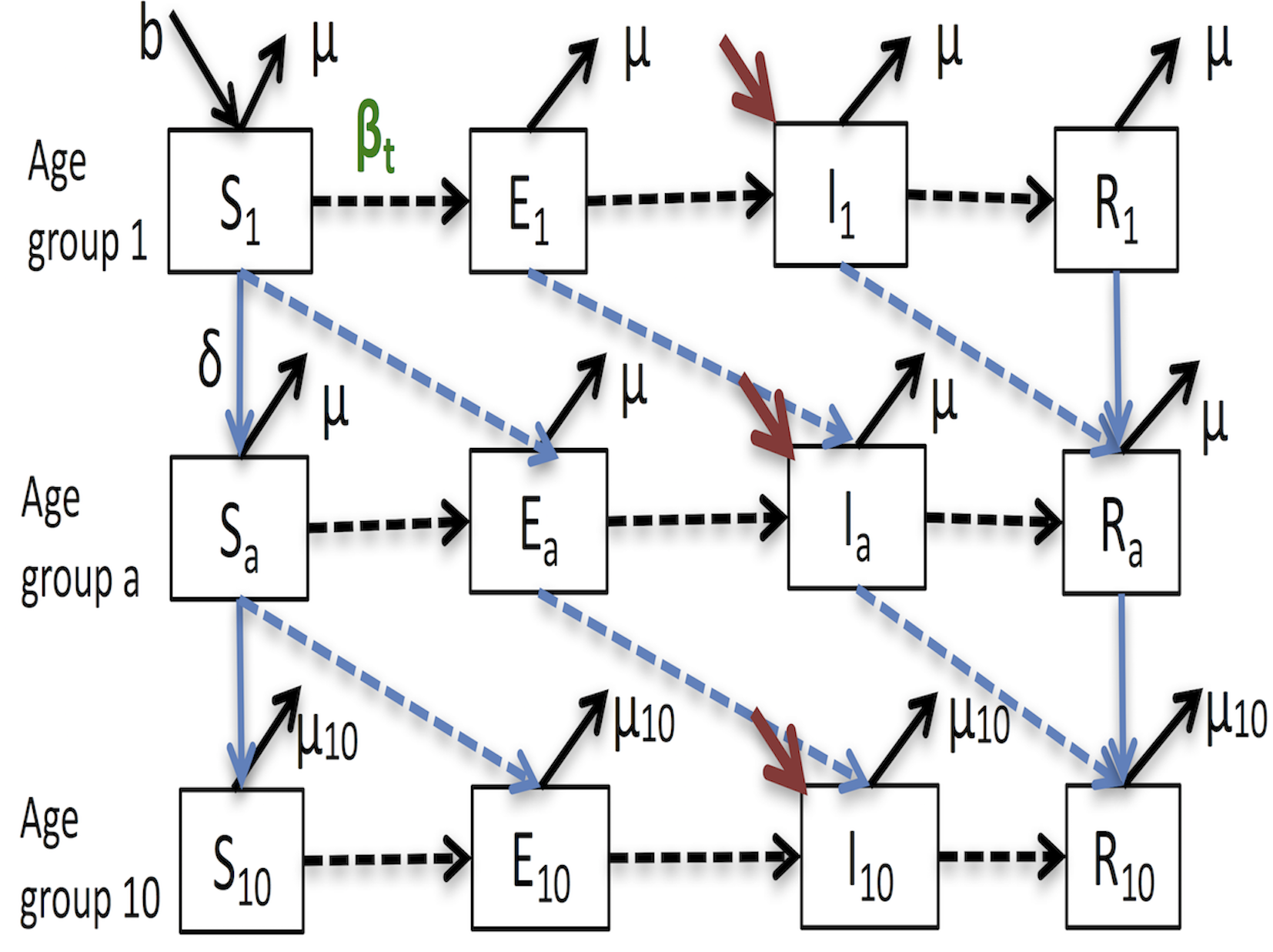

Supplement: S2 Fig — The black arrows represent the state transitions within the same yearly age group, while the blue arrows also account for the ageing of animals. The dashed lines correspond to disease stage transitions. The red arrows are the import of infectious animals, and the transmission parameter βt in green is driven by climate variables. Parameters and notations are presented in Table 1. (TIFF) [file pntd.0005767.s003.tiff]

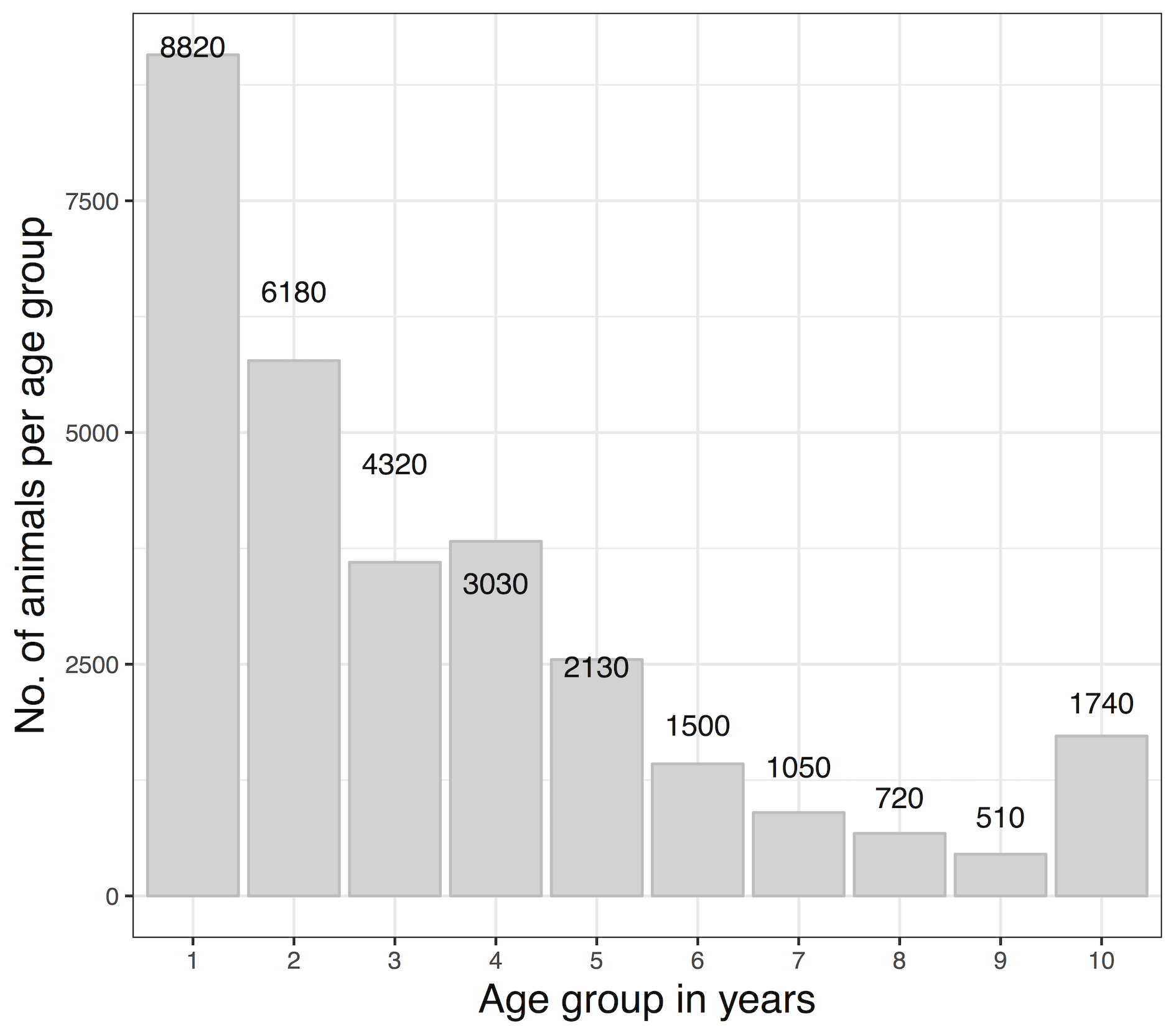

Supplement: S3 Fig — The grey bars represent the number of animals per age group according to the data na [47,48]; and the numbers written are N¯a the estimated number of animals in each age group used to parameterize the SEIR model (see S1 Text). (TIFF) [file pntd.0005767.s004.tiff]

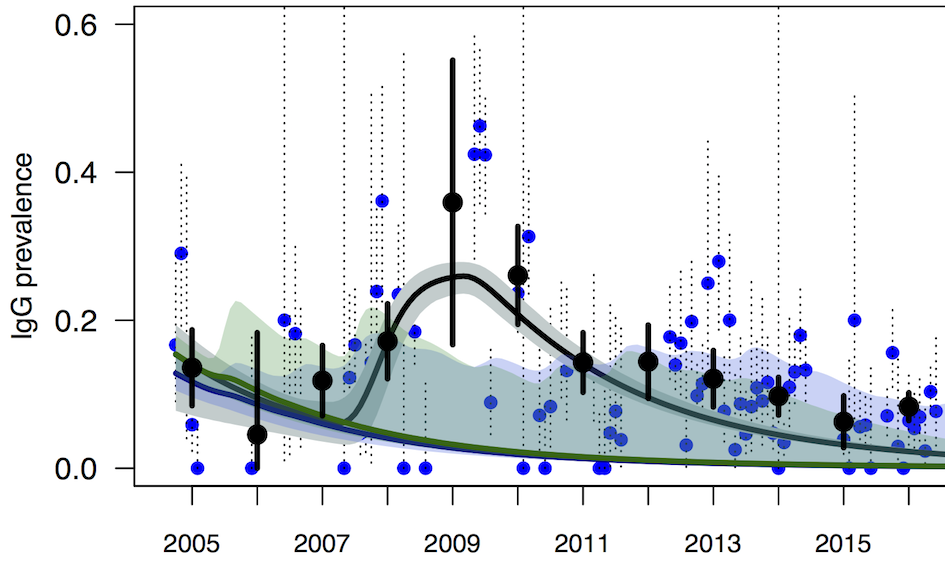

Supplement: S4 Fig — Models’ fit for the linear model (Model 2a, blue line and 95%CrI) and exponential model (Model 2b, green line and 95%CrI) assuming no animal import but allowing seasonal variations of the NDVI; and model fit with animal import but assuming no seasonal variation of the NDVI (Model 3, black line and 95%CrI). Monthly (blue dots) and annual (black dots) RVF IgG prevalence are displayed. (TIFF) [file pntd.0005767.s005.tiff]

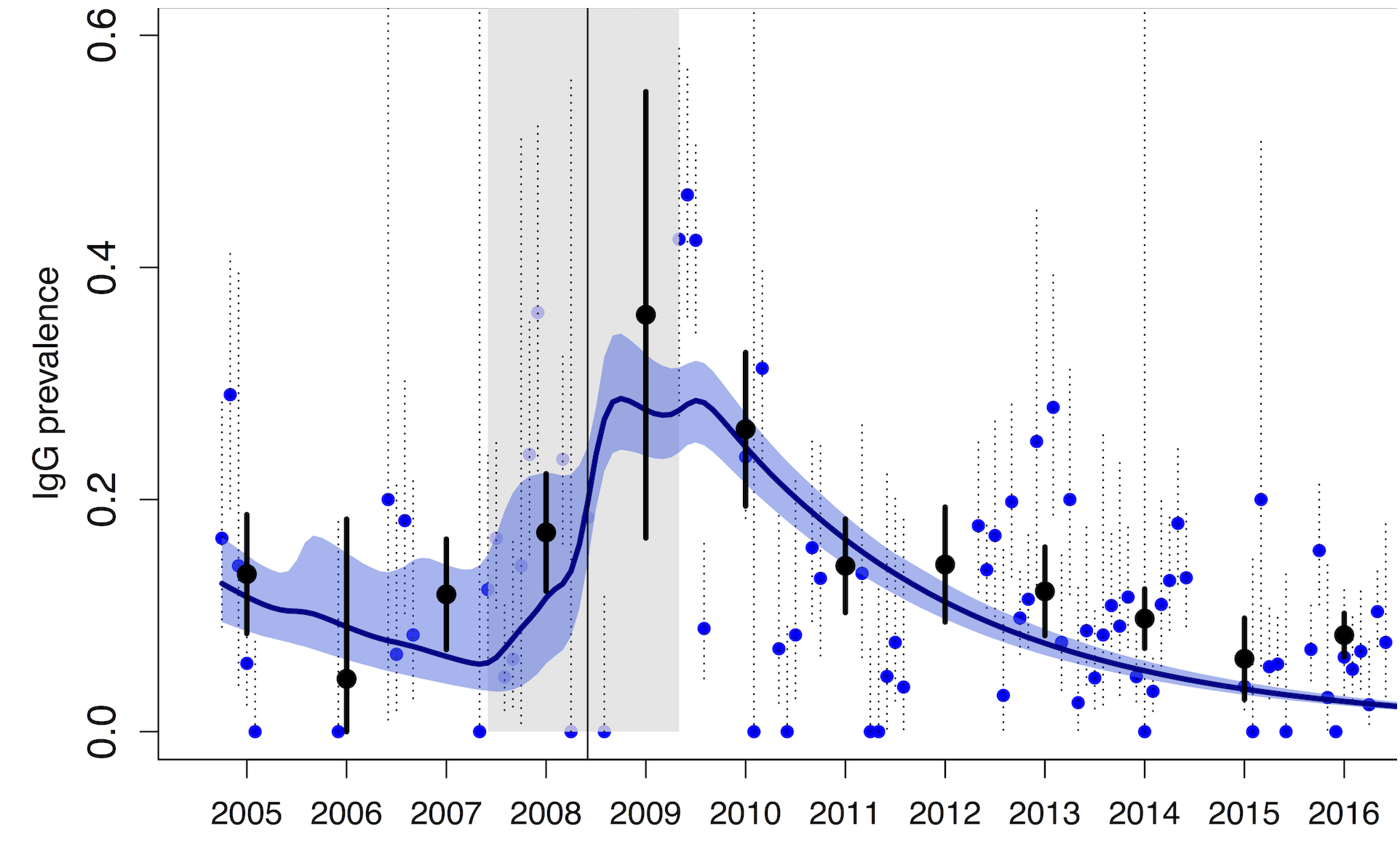

Supplement: S5 Fig — The model was fitted to the monthly seroprevalence (blue dots) for the period October 2004-June 2008 (left of the vertical black line). For the period July 2008-June 2016, the age-specific fit for each epidemiological year is shown in S6A–S6H Fig. (TIFF) [file pntd.0005767.s006.tiff]

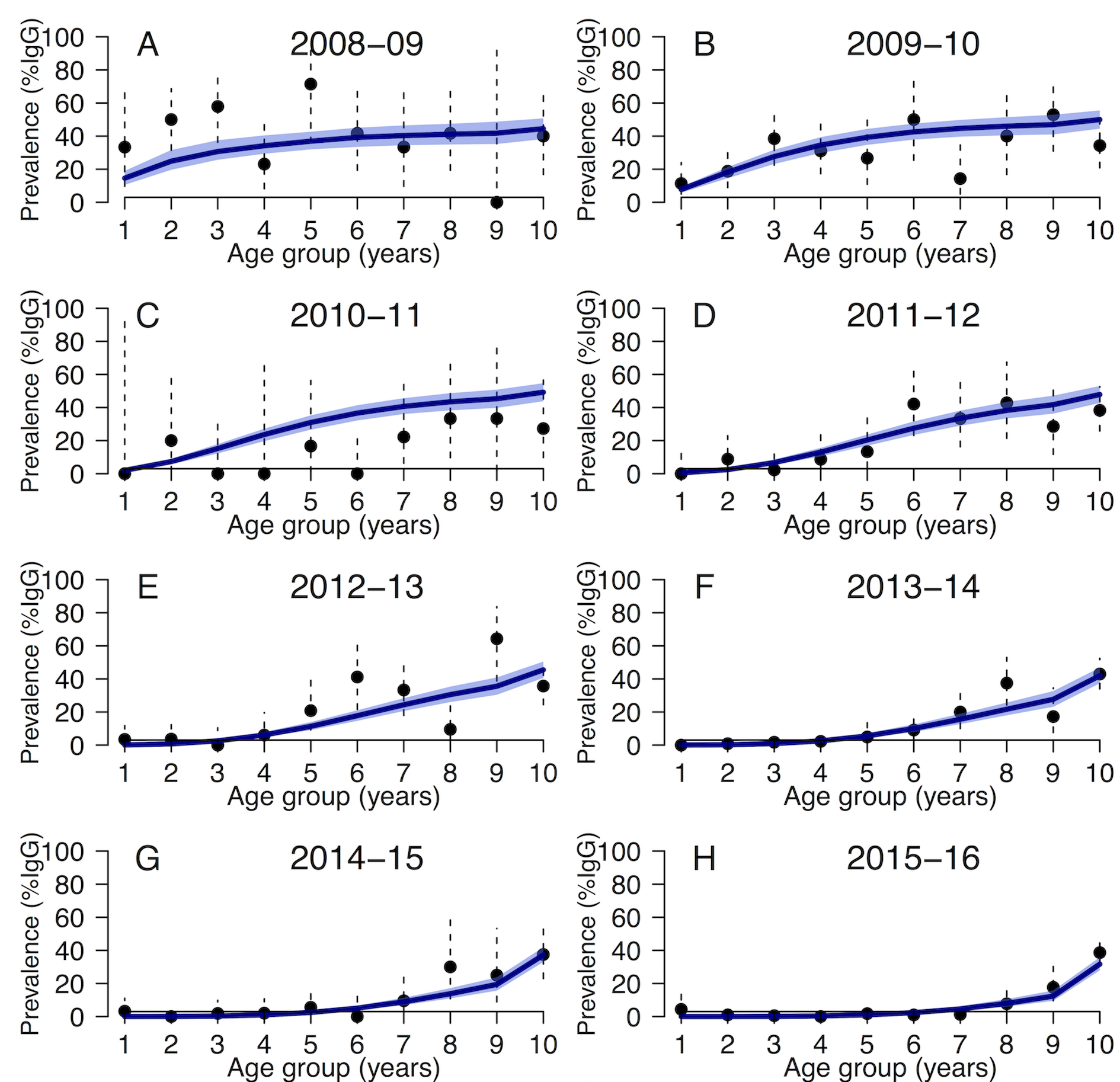

Supplement: S6 Fig — The model was fitted to the observed annual age-stratified seroprevalence (black dots) for each epidemiological year. (TIFF) [file pntd.0005767.s007.tiff]

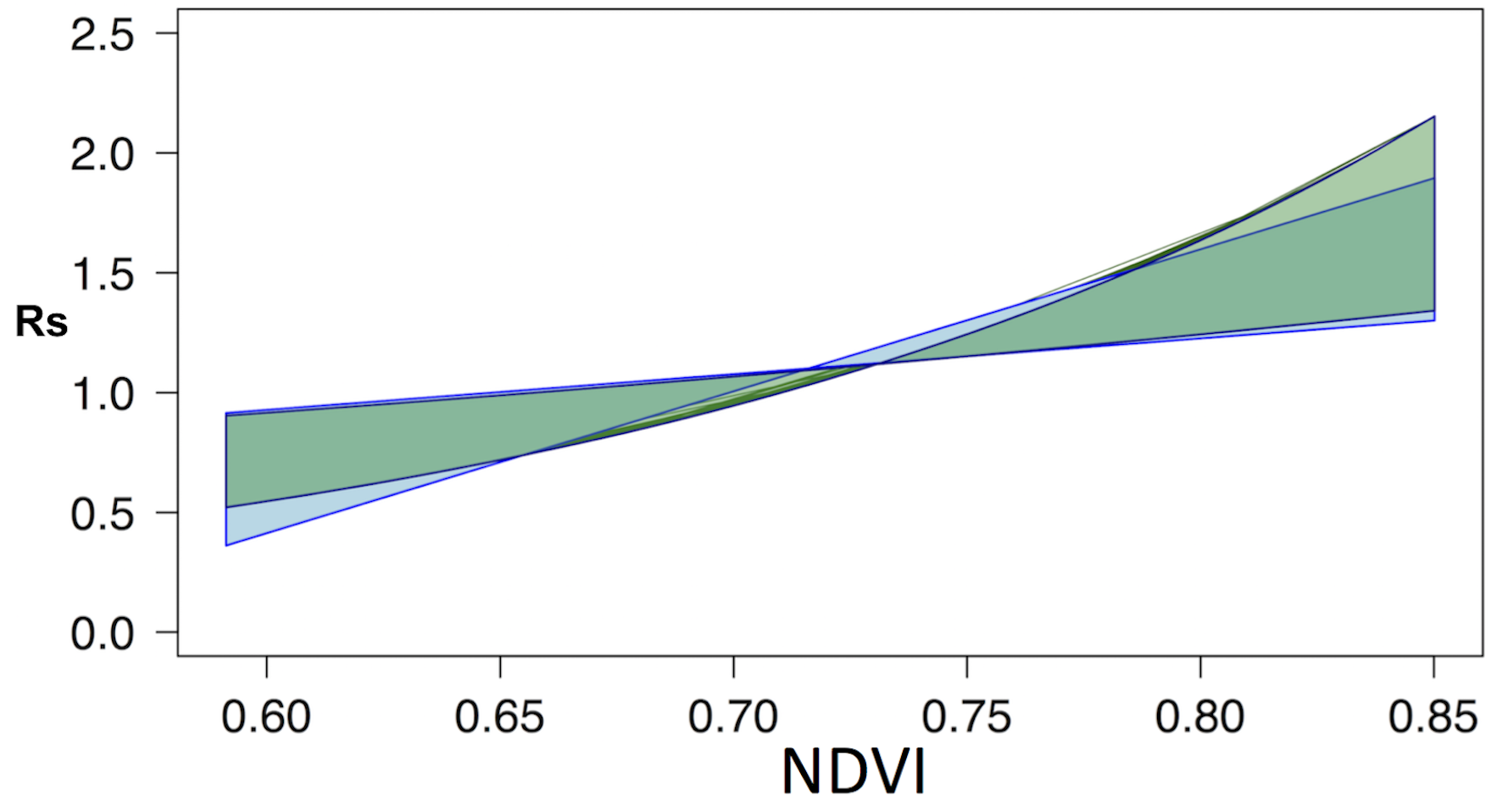

Supplement: S7 Fig — Rs values range from 0.36 to 1.90 for the linear model, and 0.52 to 2.19 for the exponential model; for NDVI values varying between 0.59 and 0.85. (TIFF) [file pntd.0005767.s008.tiff]

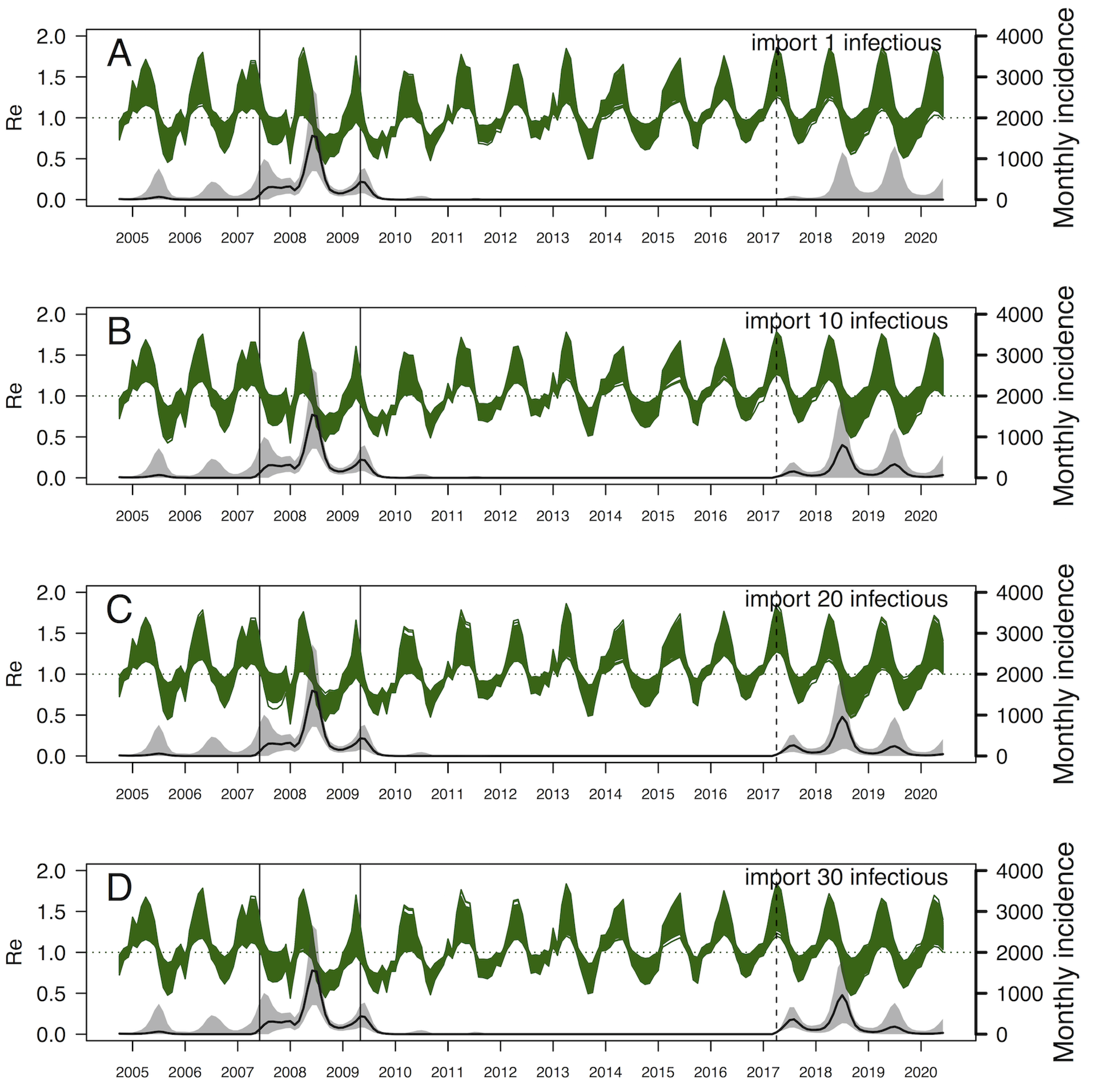

Supplement: S8 Fig — (A) Forecast 2: import of 1 infectious animals, (B) Forecast 3: import of 10 infectious animals, (C) Forecast 4: import of 20 infectious animals, and (D) Forecast 5: import of 30 infectious animals. (TIFF) [file pntd.0005767.s009.tiff]

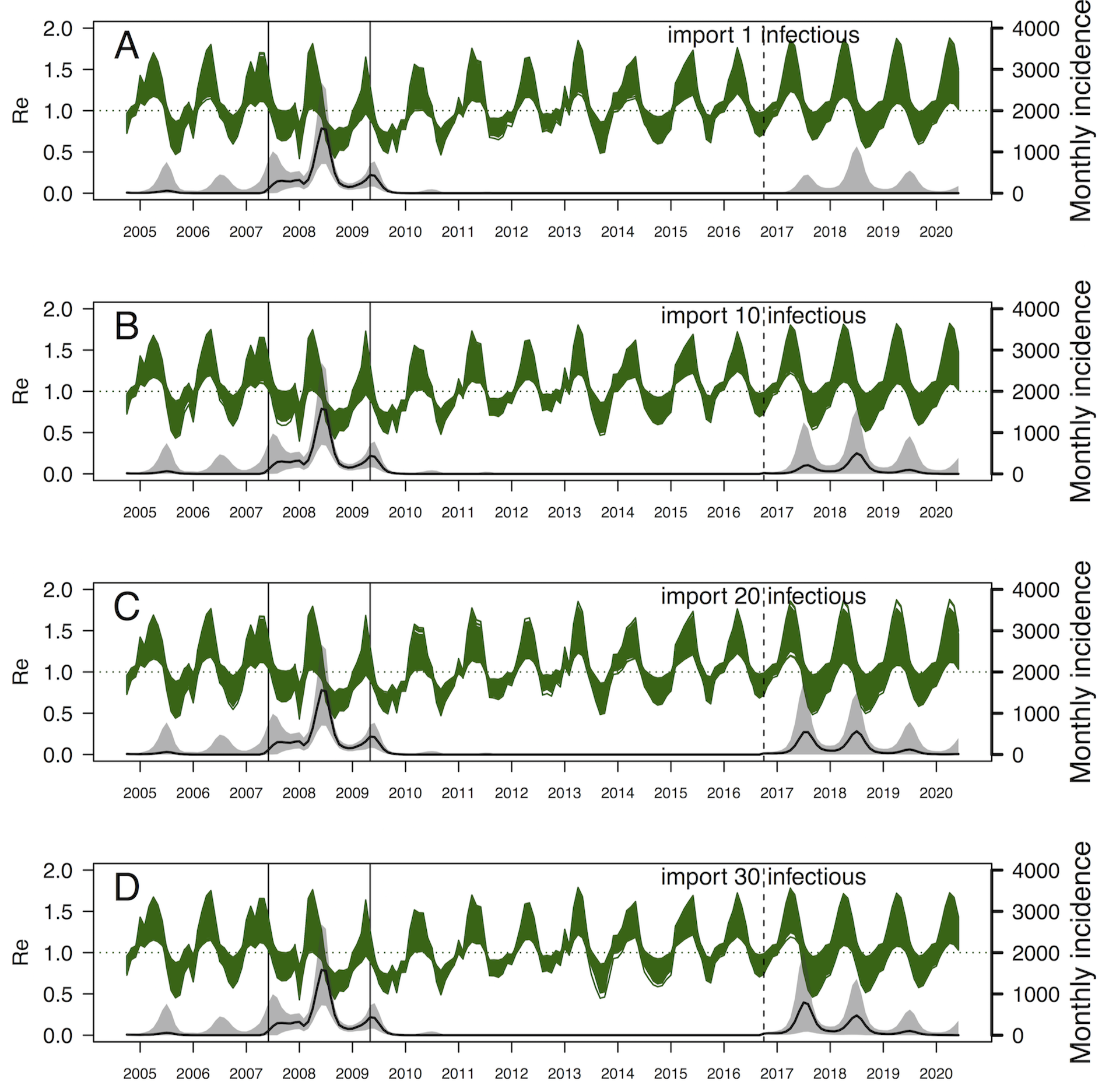

Supplement: S9 Fig — (A) Forecast 7: import of 1 infectious animals, (B) Forecast 8: import of 10 infectious animals, (C) Forecast 9: import of 20 infectious animals, and (D) Forecast 10: import of 30 infectious animals. (TIFF) [file pntd.0005767.s010.tiff]

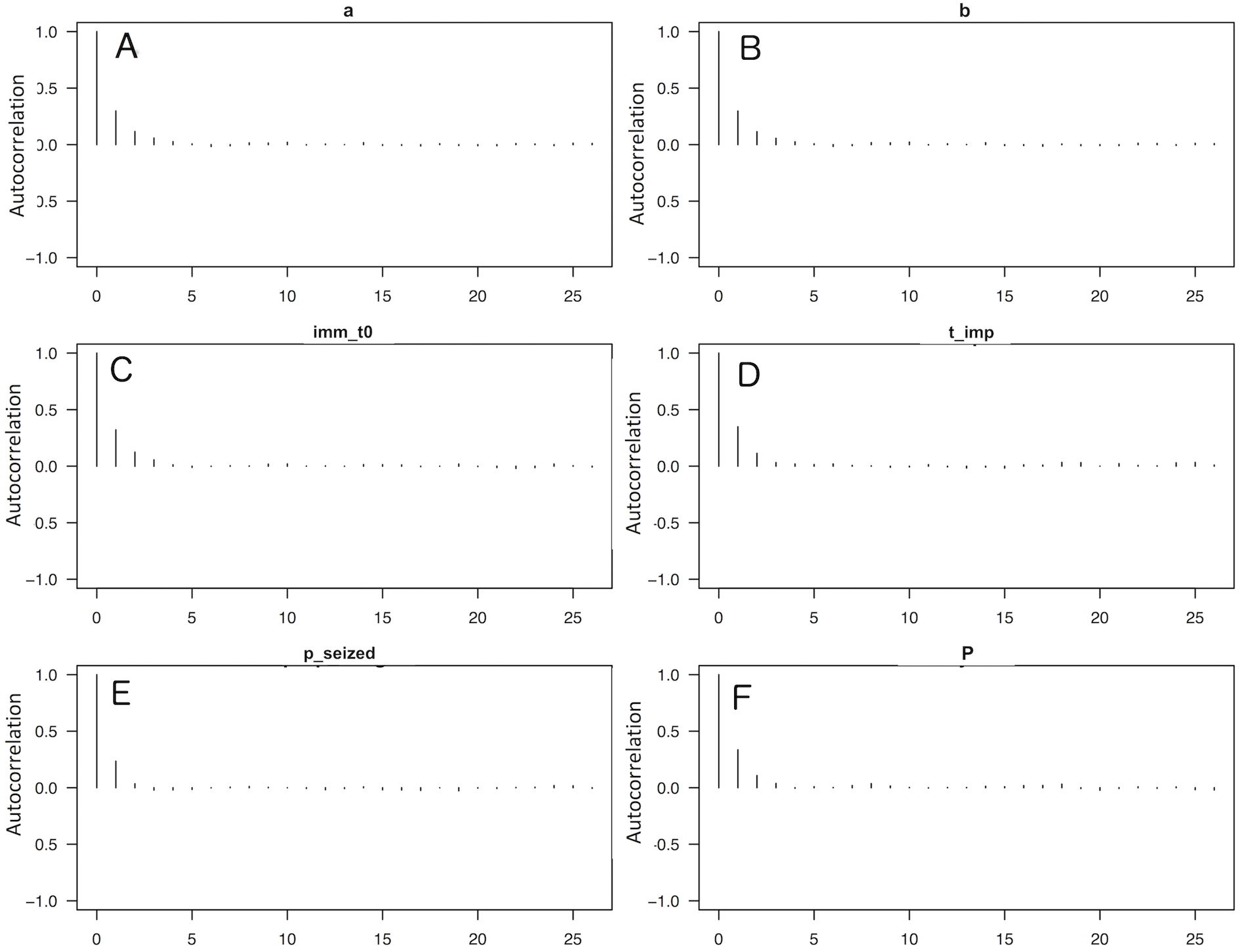

Supplement: S10 Fig — (A) multiplying factor a, (B) scaling factor b, (C) proportion of immune at t0 imm_t0, (D) date of import t_imp, (E) proportion of boats seized p_seized, (F) duration of imports P. (TIFF) [file pntd.0005767.s011.tiff]
