# Supplementary material for: Drivers for Rift Valley fever emergence in Mayotte: A Bayesian modelling approach
Source: PLoS Negl Trop Dis. 2017 Jul 21;11(7):e0005767. doi: 10.1371/journal.pntd.0005767 (PMC5540619; doi:10.1371/journal.pntd.0005767)
Supplement: S1 Table — (PDF) [file pntd.0005767.s012.pdf]

**Table S1** Demographic parameters estimated in Equations S1-S8 and used in the SEIR model

| Notation | Parameter description | Values | Source |
|---|---|---|---|
| $N$ | Total population size | 30,000 | Ref [29] |
| $N_a$ | Number of animals per age group *a* | See Fig S3 | Ref [47,48] |
| $\bar{N}_a$ | Number of estimated animals per age group a | See Fig S3 | Estimated |
| $\delta$ | Weekly ageing factor | 0.021 (1/48) | 1month=4 weeks in model |
| $\alpha_{1\text{-}9}$ | Weekly survival rate for age-groups 1 to 9 | 0.9912 | Estimated |
| $\alpha_{10}$ | Weekly survival rate for age-group 10 | 0.9938 | Estimated |
