# Supplementary material for: Drivers for Rift Valley fever emergence in Mayotte: A Bayesian modelling approach
Source: PLoS Negl Trop Dis. 2017 Jul 21;11(7):e0005767. doi: 10.1371/journal.pntd.0005767 (PMC5540619; doi:10.1371/journal.pntd.0005767)
Supplement: S2 Table — (PDF) [file pntd.0005767.s013.pdf]

**Table S2** Animal illegally entering and seized by the maritime borders in 2008 in Mayotte, and results of ELISA IgM testing (Data Veterinary Services, 2008)

| **Month** | **No. seized animals** | **No animals sampled** | **No. positive RVF IgM** |
|---|---|---|---|
| January | 3 | 0 | 0 |
| February | 4 | 4 | 4 |
| March | 2 | 2 | 1 |
| April | 20 | 19 | 3 |
| May | 18 | 14 | 2 |
| June | - | - | - |
| July | - | - | - |
| August | - | - | |
| September | 7 | 7 | 0 |
| October | 6 | 6 | 1 |
| November | 13 | 5 | 0 |
| December | 18 | 18 | 0 |
| Total | 90 | 75 | 11 |
